# Removing a Foreign Body from the Nose

**Published:** 1872-09

**Authors:** 


Removing a Foreign Body from the Nose.—Accidentally opening an old number of "Ranking's Abstract," I read an article headed, "A Novel Mode of Removing a Foreign Body from the Nose," in which is related the case of a child from whose nose surgeons failed to remove a cherry-stone, and were outdone by the village barber, who administered an emetic, and, at the moment when vomiting was about to commence, clapped a handkerchief tightly over the mouth of the child. I was reminded of the source from which was obtained a procedure I have invariably instituted in such cases, and never without success. Very many years ago, that best of practitioners, Dr. J. P. Evans (then residing in Arkansas), when on a visit to his native place, Tazewell, Tennessee, was called to the country to see a child with a foreign substance in its nostril, which had held its position in spite of effort directed by the professional skill of "all the region round." On the way, the Doctor was saluted by an aged negro woman, who asked him if he was going to see that child. On receiving an affirmative answer, she said: "Put yer finger 'long side the nose, tother side from the thing, and with yer own mouf over the child's mouf, blow hard, and it's bound to come out." He followed her directions, and occasioned the result as she had predicted. R.

Memphis, Tennessee, September, 1872.

